# Population pharmacokinetics/pharmacodynamics analysis confirming biosimilarity of SB16 to reference denosumab

**DOI:** 10.3389/fphar.2025.1631034

**Published:** 2025-08-19

**Authors:** Seungchan Choi, Suemin Park, Jinah Jung, Siook Baek, Hyeong-Seok Lim

**Affiliations:** ^1^ Department of Clinical Pharmacology and Therapeutics, Asan Medical Center, University of Ulsan College of Medicine, Seoul, Republic of Korea; ^2^ Department of Medical Science and Asan Medical Institute of Convergence Science and Technology, Asan Medical Center, University of Ulsan College of Medicine, Seoul, Republic of Korea; ^3^ Samsung Bioepis Co., Ltd., Incheon, Republic of Korea

**Keywords:** denosumab, biosimilar, target-mediated drug disposition, osteoporosis, PK/PD modeling and simulation, monolix

## Abstract

**Background:**

This analysis aims to evaluate the population pharmacokinetics (PK) and pharmacodynamics (PD) of denosumab and applied a population PK/PD approach to assess the biosimilarity of SB16 in comparison to reference product, denosumab (DEN).

**Methods:**

Pooled serum concentrations data for SB16 and DEN from male healthy volunteers (HV) in the Phase I and from postmenopausal women with osteoporosis (PMO) Phase III studies, along with lumbar spine bone mineral density (BMD) data from Phase III study, were analyzed using a nonlinear mixed effects population PK/PD sequential modeling approach. The effects of key patient variables on PK/PD parameters were assessed. Treatment effects on clearance (CL) were retained in the model, regardless of statistical significance, to enable comparative simulation between SB16 and DEN. Modeling and simulation were performed using Monolix Suite™.

**Results:**

A two-compartment target-mediated drug disposition (TMDD) model with quasi-steady state (QSS) approximation and first-order absorption adequately characterized the PK profile of denosumab. An indirect response model with maximal inhibitory function captured changes in lumbar spine BMD following treatment. The study population had a minimal effect on drug exposure and on changes in BMD, with <5% difference. Race and body weight accounted for up to 19% and 45% of the variability in drug exposure, respectively, but these differences translated into less than a 2% difference in changes in BMD for each covariate. The treatment group (SB16 vs. DEN) was not identified as a significant covariate. Including this factor on CL in the final PK/PD model, irrespective of its statistical significance, did not affect the PK/PD parameter estimates. Comparative simulations showed similar results for both treatment groups.

**Conclusion:**

The developed TMDD-QSS model with indirect response model adequately characterized the PK/PD profile of denosumab. Covariate effects, including study population (HV vs. PMO), age, and race showed no clinically meaningful impact on treatment outcomes. Covariate analysis and simulation results revealed no significant differences in PK/PD parameters between SB16 and DEN. The similarity in the PK profile and change in lumbar spine BMD between SB16 and DEN were demonstrated, supporting the potential for SB16 to be substituted for the reference product in the treatment of osteoporosis.

## 1 Introduction

Denosumab is a fully human monoclonal IgG2 antibody that binds with high affinity and specificity to receptor activator of nuclear factor kappa-Β ligand (RANKL), a critical regulator of osteoclast formation, function, and survival. By inhibiting the interaction between RANKL and its receptor RANK on osteoclast precursors and mature osteoclasts, denosumab suppresses osteoclast-mediated bone resorption, thereby increasing bone mineral density (BMD) and reducing the risk of fractures (Hanley et al., 2012; Teitelbaum, 2000).

SB16 is a biosimilar product of reference denosumab (DEN), approved in the EU under the brand names Obodence™ and Xbryk^®^ (European Medicines Agency, 2024b). In the US, SB16 is approved under the brand names Ospomyv™ and Xbryk^®^ (denosumab-dssb) (U.S. Food and Drug Administration, 2025). The similarity of SB16 to DEN was established based on totality-of-evidence data, including comprehensive evaluation of physicochemical characteristics, biological activities, and clinical performance. To support clinical similarity, pharmacokinetic (PK) bioequivalence between SB16, EU-sourced reference denosumab (EU-DEN), and US-sourced denosumab (US-DEN) was demonstrated in a Phase I, randomized, double-blind, single-dose study (SB16-1001; NCT 04621318) involving healthy male participants. Additionally, SB16 exhibited comparable pharmacodynamics (PD), safety, and immunogenicity profiles. The 90% confidence intervals (CIs) for the pairwise comparison of geometric least-squares mean ratios for the area under the concentration-time curve extrapolated to infinity (AUC_inf_), area under the concentration-time curve up to the last quantifiable concentration (AUC_last_), and the maximum serum concentration of a drug (C_max_) were all within the predefined margin of 0.80–1.25 (Lee et al., 2023). In a Phase III study (SB16-3001; NCT04664959) involving postmenopausal women with osteoporosis (PMO), clinical equivalence between SB16 and EU-DEN was demonstrated with respect to efficacy, safety, PK, PD, and immunogenicity. The 95% CI for the least-squares mean difference in percent change from baseline in lumbar spine BMD at month 12 fell within the prespecified equivalence margin, supporting comparable therapeutic effects between the two treatments (Langdahl et al., 2024).

The serum concentration-time profiles of denosumab were described using a two-compartment model with first-order absorption and parallel linear and non-linear elimination pathways. The observed non-linearity in PK is likely attributable to the binding of denosumab to RANK, which serves as a hallmark evidence of target-mediated drug disposition (TMDD). A prior population PK analysis showed that a fixed 60 mg dose produced a level of RANKL inhibition comparable to that achieved by weight-based dosing (Sutjandra et al., 2011; U.S. Food and Drug Administration, 2010; Dua et al., 2015). Denosumab PK parameters are influenced on body weight; however, the resulting differences in drug exposure did not translate into variability in the PD response (European Medicines Agency, 2010). A previous report (Marathe et al., 2011) on integrated PD models of denosumab treatment characterized the time course of BMD and bone turnover markers (BTM) using mean data reported in the literature (Hasegawa et al., 2014). To our knowledge, no formal population PK/PD analysis has been conducted using the individual level data. Therefore, the objective of our PK/PD modeling and simulation was: 1) to evaluate PK and PD characteristics of denosumab based on the pooled individual data from healthy volunteers (HV) and PMO patients, including the evaluation of key covariates influencing denosumab PK/PD; and 2) to compare the PK/PD profiles of SB16, a biosimilar, with those of the reference product, DEN.

## 2 Materials and methods

### 2.1 Study population

Population PK/PD analyses were conducted using pooled clinical data from a Phase I comparative PK study (SB16-1001) in healthy male subjects and a Phase III comparative efficacy and safety study (SB16-3001) in patients with PMO (Table 1). In the Phase I study, 168 male healthy volunteers (HV) were randomized to receive a single subcutaneous (SC) dose of 60 mg SB16, EU-DEN, or US-DEN (Lee et al., 2024). In the Phase III study, 456 PMO patients were randomized to receive either SB16 or reference EU-DEN 60 mg SC at month 0 and month 6. At month 12, patients initially treated with EU-DEN were randomized again to either continue on EU-DEN or were transitioned to SB16. Patients in the SB16 treatment group continued to receive SB16 at month 12 (Langdahl et al., 2024). The key demographic and clinical characteristics of the two study populations are summarized in Table 2. The difference in age, gender, height, and body weight among the subjects reflected the inherent distinctions between the study population (I.e., male HV vs. female PMO patients). Most subjects in both studies were Caucasian. These subject characteristics were considered during the model development process.

**TABLE 1:** Overview of SB16 comparative studies included in the population PK/PD analysis.

Study Number	Phase	Design/Objective	Treatment	Population	PK sample collection	PD sample collection
SB16-1001	I	Randomised, double-blind, three-arm, parallel group, single-dose study to compare the pharmacokinetics, pharmacodynamics, safety, tolerability, and immunogenicity of SB16 and DEN	Subjects were randomized in a 1:1:1 ratio to receive one of three treatments SB16 EU-DEN US- DEN Single-dose (60 mg) of IP were administered subcutaneously in the abdomen	Healthy male subjects N = 168^a^ (56 per treatment group)	Pre-dose and 12, 24, 48, 96, 144, 192, 240, 288, 336, 504, 672, 1008, 1344, 2016, 2688, 3360, 4,032 and 4,704 h post-dose	Not applicable
SB16-3001	III	Randomised, double-blind, multicentre clinical study to compare the efficacy, safety, pharmacokinetics, pharmacodynamics, and immunogenicity between SB16 and DEN	Main period Patients were randomized in a 1:1 ratio to receive either 60 mg of SB16 or EU-DEN subcutaneously at Months 0 and 6 Transition period At month 12, subjects who received EU-DEN in the main period were randomized again in a 1:1 ratio to either continue on EU-DEN or were transitioned to SB16 Subjects who received SB16 in the main period continued to receive SB16 (but were also followed the randomization procedure to maintain blinding)	Postmenopausal women with osteoporosis N = 457^a^ (SB16: 225 EU-DEN + SB16: 100 EU-DEN + EU-DEN: 101)	Pre-dose and 0.5, 1, 3, 6, 9, 12, 18 months after first dosing	Pre-dose, 6, 12, 18 months after first dosing

^a^ Number of subjects in the randomised set.

EU-DEN: European Union-sourced denosumab.

IP: investigational product; PD: pharmacodynamic; PK: pharmacokinetic; US: united states of americ.

PK, marker was serum concentrations of denosumab; PD, marker was lumbar spine bone mineral density.

**TABLE 2:** Summary of subject characteristics in SB16 clinical studies included in population PK/PD analysis.

Characteristics	SB16-1001 (N = 168)	SB16-3001 (N = 456)	Total (N = 624)
Continuous variables	Median (min-max)		
Age (years)	41 (28–55)	66 (52–81)	63 (28–81)
BMI (kg/m^2^)	25.75 (20.5–29.9)	24.6 (18.7–36.3)	24.85 (18.7–36.3)
Height (cm)	176 (163–197)	159 (141–180)	162 (141–197)
Weight (kg)	79.65 (60.5–94.7)	62 (47.8–89.3)	66.3 (47.0–94.7)
Categorical variables	n (%)		
Sex			
Female	0 (0)	456 (100)	456 (73.08)
Male	168 (100)	0 (0)	168 (26.92)
Race			
Asian	7 (14.58)	41 (85.42)	48 (7.69)
Black	46 (100)	0 (0)	46 (7.37)
Caucasian	115 (21.74)	414 (78.26)	529 (84.78)

BMI: body mass index; N = number of subjects; n = number of applicable subjects; Percentages are based on N in each column.

Both clinical trials were conducted in accordance with the Declaration of Helsinki (1996), the International Council for Harmonisation E6 (R2) Good Clinical Practice guidelines, and all applicable local regulatory requirements. Study protocols were reviewed and approved by the relevant Institutional Review Boards or Independent Ethics Committees at each study center. Written informed consent was obtained from all participants prior to enrollment.

### 2.2 Dataset and measurements

Overall, 6,583 serum denosumab concentration data points from 615 subjects across the Phase I and III studies, collected at prespecified timepoints in each study, along with 1,716 lumbar spine bone mineral density (BMD) measurements from 456 patients in the Phase III study, were included in the for PK/PD modeling (Table 1). In addition, a comparison of subject characteristics between DEN and SB16 in the Phase III study (SB16-3001) is provided in Supplementary Table S1.

All samples were analyzed at certified laboratories using validated analytical methods. Serum denosumab concentrations were measured employing a validated electrochemiluminescence immunoassay (ECLIA) specifically developed for the detection and quantification of denosumab. The lower limit of quantification (LLOQ) for this assay was 20 ng/mL. Among these, 615 pre-dose samples (9.34%) with concentrations below the LLOQ were set to zero for model initialization.

Of the 4,262 post-dose serum concentration samples, 3,133 samples (73.51%) were quantifiable and included in the model estimation, while 1,129 samples (26.49%) were below the LLOQ and thus treated as missing values (MDV = 1). Although excluded from the likelihood calculation, they were retained in the dataset to preserve the time structure and dosing history, ensuring accurate model fitting and simulation.

BMD of the lumbar spine (L1-4) vertebrae were measured during screening and prior to dosing at months 6, 12, and 18 using dual-energy X-ray absorptiometry (DXA) scanners from either GE Lunar [GE Healthcare, Chicago, IL, United States] or Hologic [Marlborough, MA, United States]. All scanners were certified, and DXA scan images were analyzed by the central reading center Calyx [Massachusetts, MA, United States] accordance with established practice guidelines. All patients’ subsequent DXA scans were acquired on the same scanner on which they had their baseline scan in screening. Investigator sites conducted quality control activities for DXA systems prior to the first patient scan (EMA, 2025).

### 2.3 Overall strategies for PK/PD modeling

**FIGURE 1:**
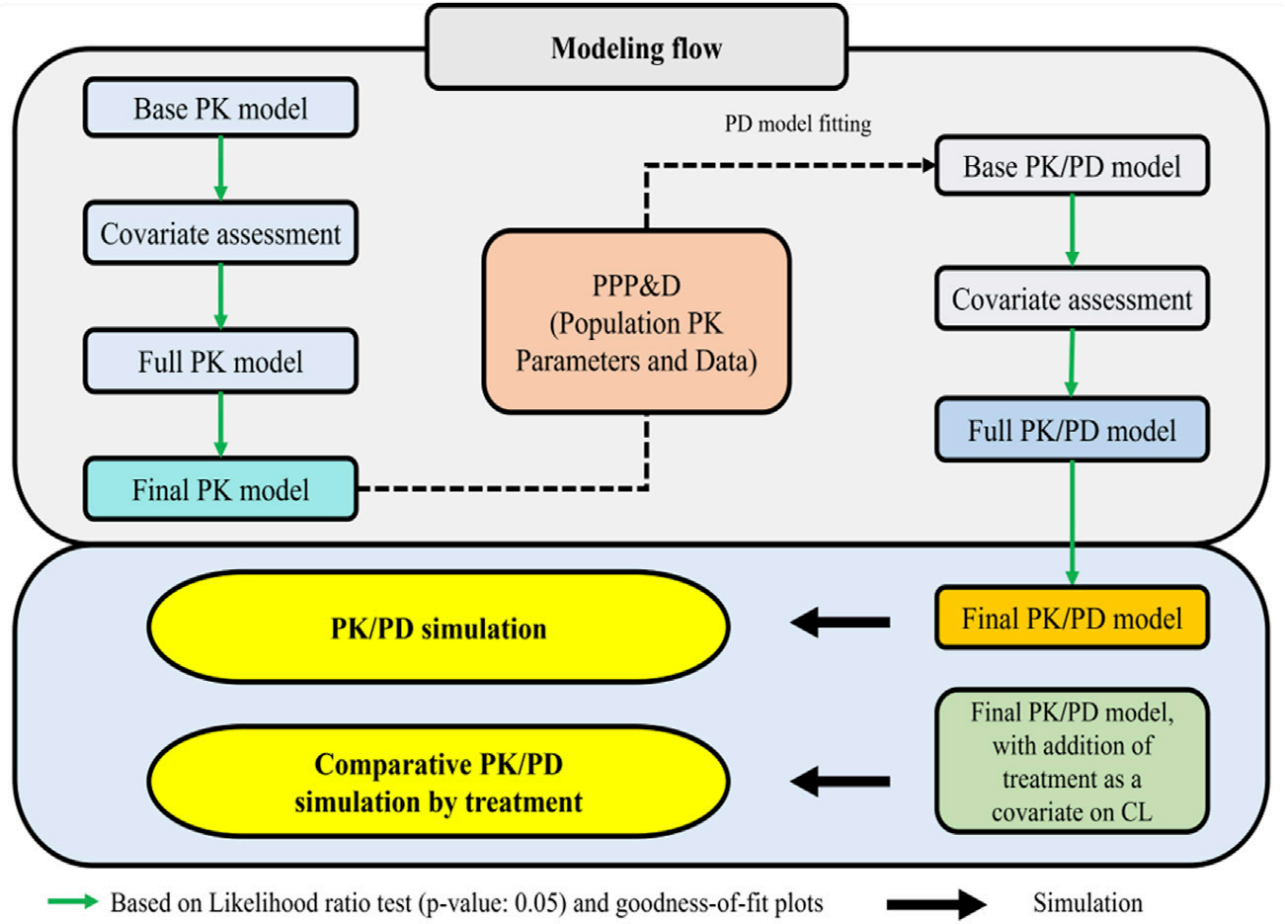
Workflow for PK/PD model and prediction of PK/PD profiles of denosumab CL: clearance; PD: pharmacodynamic; PK: pharmacokinetic: PPP&D: population PK parameters and data.

An overview of the full modeling and simulation workflow is provided in Figure 1. In summary, serum concentration data pooled from the Phase I and III studies were analyzed using a nonlinear mixed effects population modeling approach. Covariate screening was subsequently performed during PK modeling to identify significant predictors of PK parameters and to explore potential differences in PK profiles between the biosimilar, SB16 and the reference product, DEN.

PK/PD modeling was conducted in a sequential Population PK parameters and Data (PPP&D) method approach. The population PK parameters estimated from the PK model were fixed in the PK-PD model, and both PK and PD data were used simultaneously to estimate PD parameters and their IIV, as well as the individual PK parameters (Lunn et al., 2009; Lacroix et al., 2012; Zhang et al., 2003).

Covariate screening was also conducted during PD modeling to identify significant predictors of the PD outcome and to assess treatment effects (SB16 vs. DEN). In the final PK/PD model, treatment effects on clearance (CL) were intentionally included irrespective of statistical significance to enable comparative simulations between SB16 and DEN.

All PK/PD models were implemented using the stochastic approximation expectation-maximization (SAEM) method in Monolix Suite™ 2024R1 (Lixoft SAS, Antony, France), and data processing was performed using R software v4.3.1 (R Foundation for Statistical Computing, Vienna, Austria).

### 2.4 Population PK/PD structural model

**FIGURE 2:**
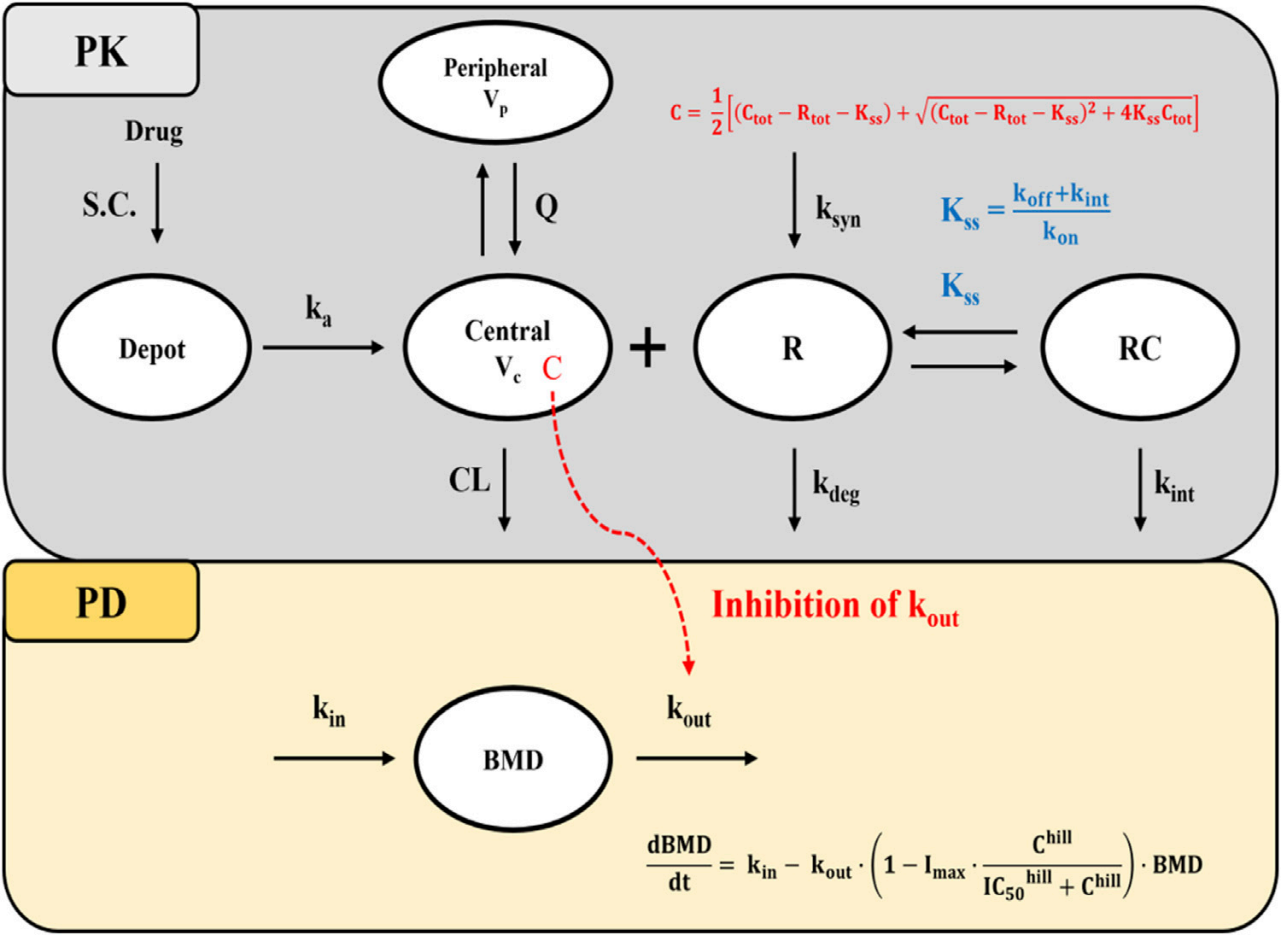
Schematic diagram of the QSS TMDD model and turnover model with inhibitory effect on k_out_. Abbreviations: S.C, subcutaneous; BMD: bone mineral density; k_a_: first-order absorption rate constant; CL: clearance; Q: inter-compartmental clearance; V_c_: volume of distribution in the central compartment; V_p_: volume of distribution in the peripheral compartment; K_ss_: quasi-steady-state equilibrium constant; k_on_: second-order association rate constant; k_off_: first-order dissociation rate constant; k_deg_: first-order target degradation rate constant; k_int_: first-order internalization rate constant of the drug-target complex; k_syn_: zero-order target synthesis rate constant; IC_50_: drug concentration that produces 50% of the maximum inhibition; I_max_: Maximum inhibitory effect of drug; k_in_: zero-order rate constant for BMD synthesis; k_out_: first-order rate constant for BMD elimination; hill: hill coefficient; C:free drug concentration; C_tot_: total drug concentration; R_tot_: total target receptor concentration; Note that R and RC compartments represent the total target receptor activator of nuclear factor kappa-B ligand (RANKL) and RANKL-drug complexes, respectively.

Consistent with the known PK characteristics of denosumab, exploratory analysis of serum concentration-time profiles following administration of SB16 and EU/US-DEN exhibited target-mediated drug disposition (TMDD) (Sutjandra et al., 2011; Gabrielsson et al., 2016). Accordingly, one and two-compartmental TMDD models with several approximations to drug-target receptor binding kinetics were tested (Dua et al., 2015; Gibiansky et al., 2008), and a two-compartment, quasi-steady-state (QSS) approximation of the TMDD model best described the PK data (Figure 2). The amount of drug administered subcutaneously ( $A_{sc}$ ) into the depot, the total (free and bound) concentration of the drug ( $C_{tot}$ ) in the central compartment, the amount of free drug in the peripheral (*A*_*p*_) compartment, and total concentration of free and bound target RANKL ( $R_{tot}$ ), as defined by the QSS model, are described by respective differential Equations 1–4:

$$\frac{dA_{\text{SC}}}{\text{dt}}=-k_{a}\cdot A_{\text{SC}}$$ (1)

$$\frac{dC_{\text{tot}}}{\text{dt}}=\frac{k_{a}\cdot A_{\text{SC}}}{V_{C}}-\left(\text{CL}+Q\right)\cdot C-\frac{K_{\mathrm{int}}\cdot R_{\text{tot}}\cdot C}{K_{\text{ss}}+C}+Q\;\cdot \frac{A_{p}}{V_{p}}$$ (2)

$$\frac{dA_{p}}{\text{dt}}=Q\;\cdot \left(C-\frac{A_{p}}{V_{p}}\right)$$ (3)

$$\frac{dR_{\text{tot}}}{\text{dt}}=k_{\text{syn}}-k_{\deg}\cdot R_{\text{tot}}-\left(k_{\mathrm{int}}-k_{\deg}\right)\cdot \frac{R_{\text{tot}}\cdot C}{K_{\text{SS}}+C}$$ (4)

In the absence of drug target level data, it was assumed that the free drug, target, and drug-target complex are in a QSS, where the binding rate is balanced by the sum of dissociation and internalization rates. This is described by steady-state constant $K_{ss}$ = $\frac{k_{\mathrm{int}}+k_{off}}{k_{on}}$ (Dua et al., 2015; Gibiansky et al., 2008).

Under QSS conditions, the concentration of free drug (C) and drug-target complexes (RC) are described using Equation 5–6:

$$\text{RC}=\frac{R_{\text{tot}}\cdot C}{K_{\text{ss}}+C}$$ (5)

$$C=\frac{1}{2}\left[\left(C_{\text{tot}}-R_{\text{tot}}-K_{\text{ss}}\right)+\sqrt{{\left(C_{\text{tot}}-R_{\text{tot}}-K_{\text{ss}}\right)}^{2}+4\cdot K_{\text{ss}}\cdot C_{\text{tot}}}\right]$$ (6)

Prior to drug administration, it was assumed that the turnover rate of free target receptors ( $R_{0}$ ) was at steady-state, such that ${k_{syn}=R}_{0}\times$ $k_{\deg}$ (Equation 7), and no drug-bound target was present in the system at baseline. The baseline receptor concentration (*R*_*0*_), QSS constant (*K*_*SS*_), and internalization rate constant (*k*_*int*_) were estimated from the observed PK data under QSS assumptions.

$$R_{0}=\frac{k_{syn}}{k_{\deg}}$$ (7)

For the PD model, an indirect response model based on turnover kinetics was employed to describe the changes in BMD following drug administration. Based on the mechanism of action of denosumab which reduces bone resorption by preventing formation and activity of osteoclast the inhibition of the elimination rate of lumbar spine BMD ( $\left.k_{out}\right)$ was selected from among various indirect response models evaluated during PD model development (Dayneka et al., 1993). The inhibition effect was modeled using a sigmoid maximum effect (*I*_max_) function, and the hill coefficient (*hill*) was estimated as a model parameter to characterize the steepness of the concentration-response relationship, as described in Equation 8 (Gabrielsson et al., 2007):

$$\frac{\text{dBMD}}{\text{dt}}=k_{\text{in}}-k_{\text{out}}\cdot \text{BMD}\cdot \left(\;1-\frac{I_{\max}\cdot C^{\text{hill}}}{{{\text{IC}}_{50}}^{\text{hill}}+C^{\text{hill}}}\right)$$ (8)

Prior to drug administration, it was assumed that the BMD turnover system was at steady state, such that the BMD production rate constant (kin) equals the product of the BMD elimination rate constant (kout) and the baseline BMD (BMD_0_), as shown in Equation 9:

$$k_{in}=k_{out}\;\cdot {BMD}_{0}$$ (9)

Under this assumption, *k*_*in*_ was not directly estimated but calculated accordingly.

To confine the estimated *I*_max_ parameter within the biologically plausible range of 0 and 1, and to implement interindividual variability on *I*_max_, a logit transformation was applied as shown in Equation 10 (Adedokun et al., 2020):

$$I_{\max}=\frac{e^{I_{\max}F}}{\left(1+e^{I_{\max}F}\right)}$$ (10)

where I_max_F is an unbounded parameter representing the logit-transformed value of I_max_.

### 2.5 Statistical model

Interindividual variability (IIV) in most PK/PD parameters was modeled using an exponential distribution (Equation 11). The IIV for *i*th individual, *η*_*i*_, is a random variable assumed to be independently selected from normal distribution with a mean of zero and variance of ω^2^.

$$P_{i}=P_{TV}\times \exp\left(\eta_{i}\right)$$ (11)

where $P_{TV}$ : typical value (population value) of the fixed effect parameter

The residual error model describing the difference between observed and the model-predicted values was selected based on the likelihood ratio test (LRT) and inspection of goodness-of-fit (GOF) plots.

Based on the analysis, a combined error model (Equation 12) was selected to describe the residual error in serum denosumab concentration, and an additive error model (Equation 13) was selected for lumbar spine BMD. The residual variability (RV) for the *j*th observed value in the *i*th individual, ε_add,ij_, ε_prop,ij_, represent additive and proportional components, respectively. These errors are assumed to be independent and normal distribution, each with a mean of zero and a variance of σ^2^.

$$y_{ij}={IPRED}_{ij}\times \left(1+\varepsilon_{prop,ij}\right)+\varepsilon_{add,ij}$$ (12)

$$y_{ij}={IPRED}_{ij}+\varepsilon_{add,ij}$$ (13)

where y_ij_: *j*th observed value in the *i*th individual; IPRED_ij_: *j*th model predicted value in the *i*th individual; ε_add,ij_ additive error; ε_prop,ij_ proportional error.

### 2.6 Model selection and evaluation

Model discrimination and validation were performed using statistical and graphical approaches. For statistical methods, the LRT was used to differentiate between hierarchical models at p-value <0.05, considering that the difference of −2 log-likelihood (-2LL) of the model follows an approximate chi-square distribution. A p-value of 0.05, corresponding to a decrease in objective function value (OFV) of 3.84 points, was considered as statistically significant. For the Wald test, a 95% CI for each parameter estimate was constructed using the point estimate and standard error derived from the model outcomes and checked if 95% CI excluded 0.

Basic GOF plots and visual predicted checks (VPCs) were applied to compare the observed values and model-predicted values, and model prediction was evaluated in terms of central tendency and variability. VPCs were performed by simulating 1000 replicates, and the resulting simulations were overlaid and compared with the original PK/PD data in plots generated using Monolix.

### 2.7 Covariate assessment

Covariate selection was performed using the conditional sampling use for the stepwise approach based on the correlation (COSSAC) algorithm, as implemented in Monolix. Study population (HV vs. PMO patients), body weight, age, race, and treatment group (biosimilar SB16 vs. DEN) were tested on relevant PK/PD parameters. At each iteration for covariate screening, covariates were checked for addition at p-value of 0.01 corresponding to a 6.635 points difference in log-likelihood (LL) then removal at p-value of 0.001 corresponding to 10.828 points difference in LL. The procedure alternated between forward inclusion and backward elimination steps, and the final model was selected based on the LRT (Ayral et al., 2021).

Continuous covariates were incorporated into the model using a normalized power function centered at the median value, and categorical covariates were modeled using an exponential function as presented in Equations 14,15, [Disp-formula e15]respectively:

$$TVP_{i}=TVP_{ref}\cdot {\left(\frac{Cov_{i}}{Cov_{ref}}\right)}^{\theta }$$ (14)

$$TVP_{i}=TVP_{ref}\cdot e^{\theta \cdot Cat_{i}}$$ (15)

where TVP_i_ is the typical value for *i*th individual, Cov_i_ is the *i*th individual covariate value, Cov_ref_ is the reference (median) value, θ is the estimated covariate effect, and Cat_i_ is a binary indicator variable representing the categorical covariate (1 for tested variable, 0 for reference).

The final covariate model was automatically selected by the COSSAC algorithm as the model with the lowest −2 log-likelihood among the candidate models explored through stepwise forward and backward procedures. After selection, the numerical stability of parameter estimates (e.g., standard errors and relative standard errors) was evaluated, and covariates associated with unstable estimates were removed.

### 2.8 Model-based simulation

Monte-Carlo simulations of serum denosumab concentration-time and lumbar spine BMD-time profile were performed by the treatment group and covariate subgroups using Simulx (Monolix Suite™ 2024R1). The simulations were based on the final population PK/PD models. For each of SB16 and EU/US-DEN treatment groups, simulation data were generated for 1,000 virtual participants receiving three successive SC administrations of 60 mg of drug at 6-month interval. The IIV and RV were incorporated to reflect population variability. Covariates were fixed based on predefined subgroup scenarios rather than sampled from distributions.

The fifth, 50th (median), and 95th percentiles of the PK metrics were calculated and compared across subgroups identified as significant predictors of PK during covariate assessment. Similarly, the percentage change from baseline in lumbar spine BMD was calculated and summarized using the median and 90% prediction interval (PI) for each subgroup.

### 2.9 PK/PD comparison between treatments

The treatment effects of the biosimilar SB16 and reference DEN, pooled from EU/US-DEN data, on the CL and I_max_ were assessed during covariate analysis. Furthermore, empirical Bayes estimates (EBEs) for key PK and PD parameters were visually compared between the two treatments using boxplots.

For the simulations used to compare PK and PD profiles of SB16 and DEN, the treatment group was intentionally retained as a covariate in the final PK/PD model, irrespective of its statistical significance in the covariate analysis. For each subject, PK metrics such as C_max_, time to peak drug concentration (T_max_) and area under the concentration-time curve within a dosing interval (τ) at steady state (AUC_τ,ss_) and change from baseline in lumbar spine BMD were calculated from the simulation data. The median and 90% PI were then summarized and compared between the two treatment groups.

## 3 Results

### 3.1 PK/PD model

The PK of denosumab was well-described by a two-compartment TMDD model with QSS approximation, adequately characterizing data from the Phase I study in healthy male volunteers and the Phase III study in female PMO patients (Table 3). IIV was estimated for all PK parameters. Statistically significant correlation between IIVs of CL/F and V_P_/F was identified after testing the correlations among all the IIVs. The PD model was described using an indirect response model with inhibitory effect on the elimination process (Table 4). Including IIV on the baseline BMD (BMD_0_) and the IC_50_ significantly improved the model fit.

**TABLE 3:** Final population pharmacokinetic parameter estimates of denosumab.

Parameter (units)	Definition	Estimate [%RSE]	Inter-individual variability [%RSE]
k_a_ (1/h)	Absorption rate constant for healthy subjects	0.014 [4.56]	56.57 [5.05]
k_a__PMO (1/h)	Absorption rate constant for PMO patients	0.0078 [4.96]	
V_C_/F (L)	Volume of the central compartment	1.58 [4.30]	61.69 [5.68]
Covariate effect (θ) of body weight on V_C_/F	Covariate effect (θ) in the power model is described as $V_{C}/F\times {\left(\frac{WT}{64}\right)}^{\theta }$	1.50 [13.78]	-
V_P_/F (L)	Volume of the peripheral compartment	6.06 [1.20]	15.55 [6.65]
Covariate effect (θ) of body weight on V_P_/F	Covariate effect (θ) in the power model is described as $V_{P}/F\times {\left(\frac{WT}{64}\right)}^{\theta }$	0.52 [11.25]	-
k_int_ (1/h)	Denosumab-target complex internalization rate constant	0.022 [1.71]	7.88 [13.46]
K_SS_ (nmol/L)	Quasi-steady state equilibrium constant	1.56 [12.41]	58.12 [23.07]
k_syn (_1/h)	target (RANKL) synthesis rate constant	0.01 [2.86]	22.46 [11.92]
R_0_ (nmol/L)	Baseline target (RANKL) for HV	0.98 [24.33]	158.3 [7.14]
R_0__PMO (nmol/L)	Baseline target (RANKL) for PMO	15.23 [12.78]	
CL/F in Caucasian (L/h)	Clearance for Caucasian	0.006 [1.46]	26.39 [3.67]
CL/F on Black (L/h)	Clearance for Black	0.0069 [4.15]	
CL/F on Asian (L/h)	Clearance for Asian	0.0074 [3.96]	
Covariate effect (θ) of body weight on CL/F	Covariate effect (θ) in the power model is described as $CL/F\times {\left(\frac{WT}{64}\right)}^{\theta }$	0.93 [7.98]	-
Q/F (L/h)	Inter-compartment clearance for HV	1.13 [15.57]	295.99 [6.97]
Q/F_PMO (L/h)	Inter-compartment clearance for PMO	0.20 [20.22]	
Correlation			
CORR_Vp/F-CL/F_	Correlation between IIV of CL/F and IIV of Vp/F	0.43 [12.35]	
Residual error			
ε_add_ (nmol/L)	Additional error	0.72 [4.05]	-
ε_prop_	Proportional error	0.07 [3.74]	-

Inter-individual variability (IIV) is expressed as coefficient of variation (CV %); CORR: correlation; PMO: postmenopausal osteoporosis; HV: healthy volunteer; RSE: relative standard error.

**TABLE 4:** Final population pharmacodynamic parameter estimates of denosumab.

Parameter (units)	Definition	Estimate [%RSE]	Inter-individual variability [%RSE]
BMD_0_ (g/cm^2^)	Baseline Lumbar spine BMD	0.76 [0.46]	59.85 [3.37]
k_out_ (1/h)	Rate constant for the elimination of Lumbar spine BMD	0.00018 [9.5]	-
k_in_ (g/cm^2^/h)	Rate constant for the production of Lumbar spine BMD	0.00014^a^	
I_max_F	Logit-transformed fractional maximum inhibitory effect	−1.75 [3.02]	-
IC_50_ (nmol/L)	Inhibition effect achieving 50% of Imax	6.92 [11.65]	9.52 [17.45]
HILL	Hill coefficient	0.17 [4.9]	-
Residual error			
ε_add_ (g/cm^2^)	Additional error	0.02 [2.06]	-

Inter-individual variability (IIV) is expressed as coefficient of variation (CV %); RSE: relative standard error.

^a^ The production rate constant (k_in_) was not estimated directly, but calculated under the assumption of baseline steady state (i.e., C = 0), using the relationship k_in_ = k_out_ x BMD_0_.

All the fixed-effect PK/PD parameters and random effect parameters for IIV were estimated with acceptable precision, as indicted by relative standard errors (RSEs) < 25%. Although IIV of inter-compartmental clearance (Q) in healthy subjects was estimated to be high (coefficient of variation, CV: 295.99%), the estimate of the IIV was also obtained with high precision (RSE: 6.97%), suggesting that the observed variability is supported by the data. No significant signs of parameter instability or over-parameterization were observed.

Covariate analysis revealed that the study population (HV vs. PMO patients) statistically significantly affected k_a_ (ratio of HV/PMO: 0.55), R_0_ (ratio: 15.49), and Q (ratio: 0.18). Body weight influenced V_C_/F, V_P_/F, and CL/F through a power model, with exponents of 1.5, 0.93, and 0.52, respectively. Race influenced CL/F, with values 1.14 times higher in Black individuals and 1.22 times higher in Asians compared to those of Caucasians. No patient-related variables, including treatment groups, were identified as covariates for any PD parameter.

**FIGURE 3:**
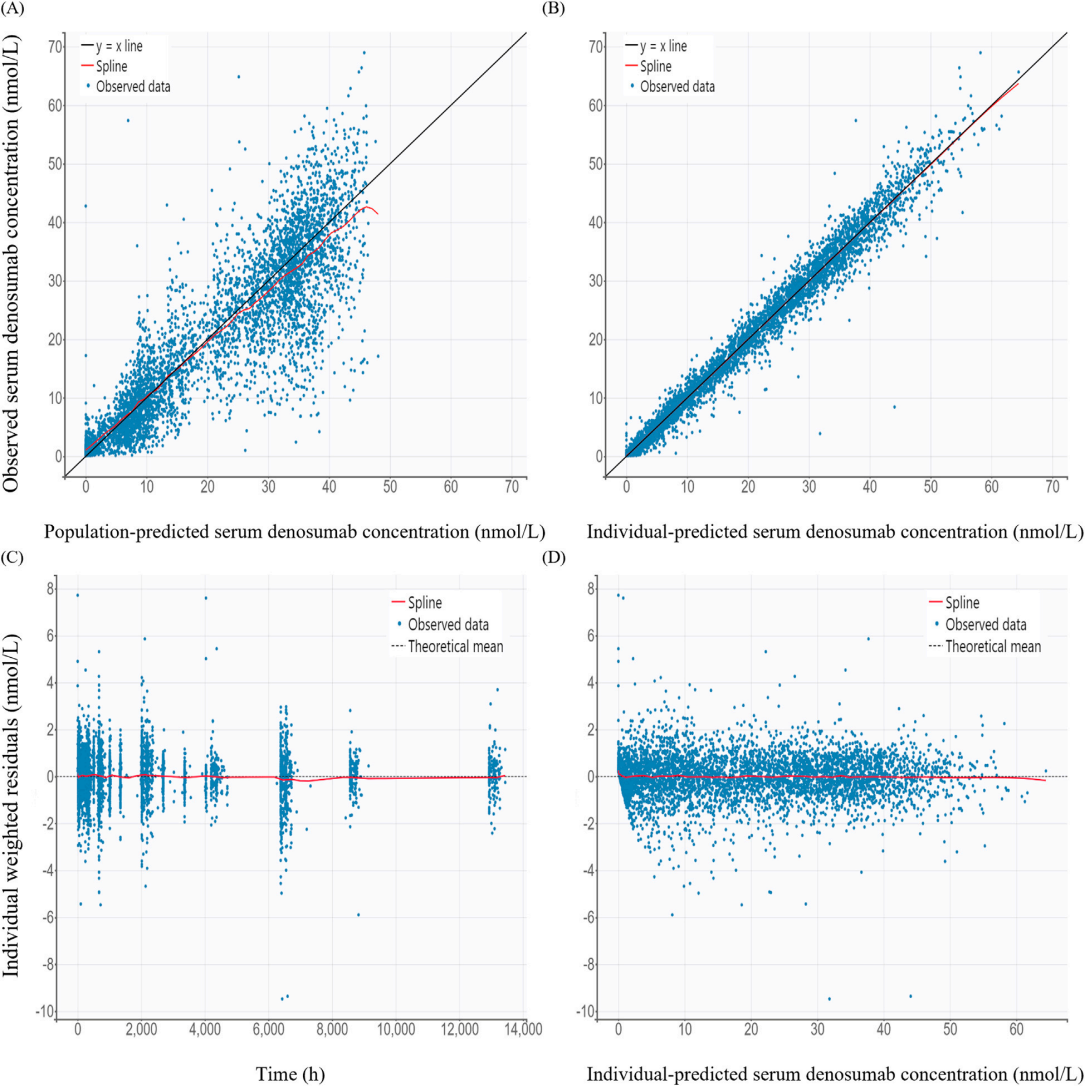
Basic goodness-of-fit plots of the final pharmacokinetic model **(A)** Observed versus population-predicted concentration; **(B)** Observed versus individual-predicted concentration; **(C)** Individual weighted residuals versus time; **(D)** Individual weighted residuals versus individual-predicted concentration.

**FIGURE 4:**
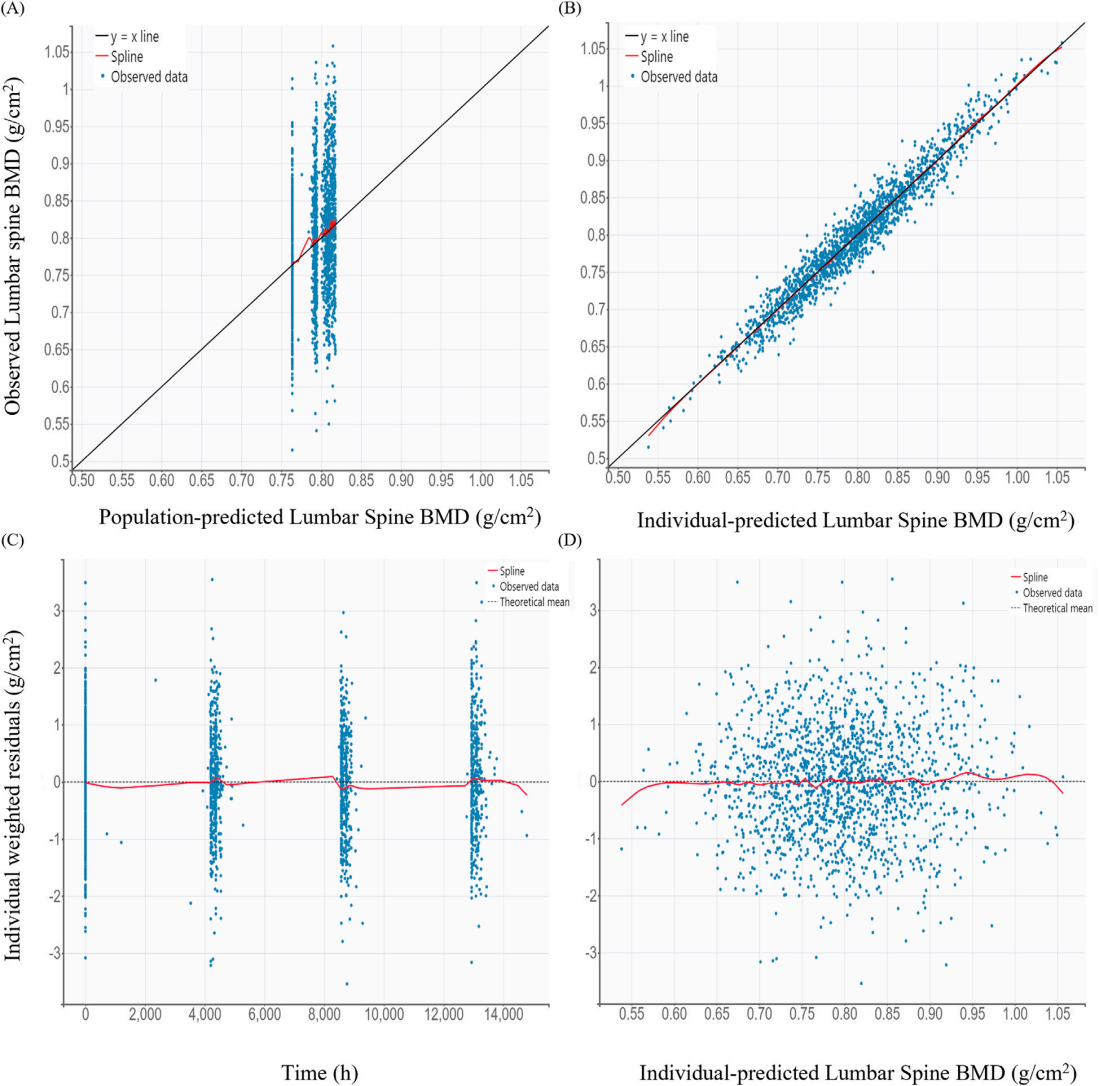
Basic goodness-of-fit plots of the final pharmacodynamic model **(A)** Observed versus population-predicted lumbar spine bone mineral density (BMD); **(B)** Observed versus individual-predicted lumbar spine BMD; **(C)** Individual weighted residuals versus time; **(D)** Individual weighted residuals versus individual-predicted lumbar spine BMD.

GOF plots for the PK/PD model demonstrated good agreement between observed and predicted values (Figures 3, 4). The individual prediction plots (IPRED) showed data points closely clustered around the line of identity, indicating that the model adequately captured IIV (Figures 3B, 4B). Residual diagnostics further supported model adequacy, as individual weighted residuals (IWRES) were randomly distributed around zero across both time and predicted concentration axes. No patterns or evidence of heteroscedasticity were observed (Figures 3C,D, 4C,D).

**FIGURE 5:**
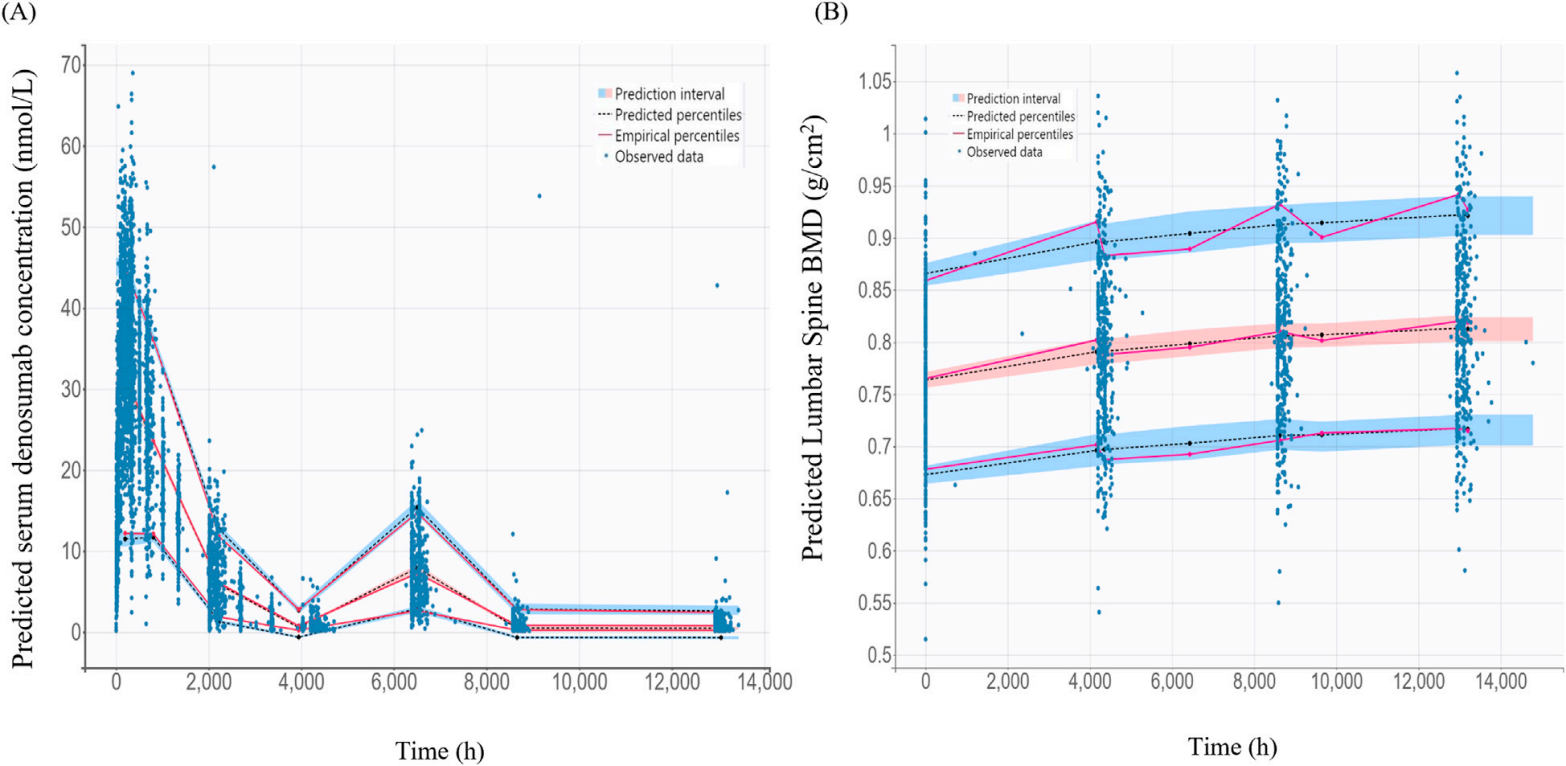
Visual predictive check for the final pharmacokinetic model and pharmacodynamics models **(A)** Final pharmacokinetic model for denosumab concentration; **(B)** Final pharmacodynamic model for lumbar spine bone mineral density (BMD); The black dashed lines represent the 5%, 50%, 95% percentiles of the model predictions. The shaded blue areas indicate the 95% prediction intervals for each percentile. Observed data are as blue dots.

The VPC for the final PK/PD model showed that model predictions closely aligned with the observed data for serum denosumab concentrations and BMD (Figure 5). Most observed values fell within the 90% PI, indicating strong predictive performance.

### 3.2 Model-based simulations

**FIGURE 6:**
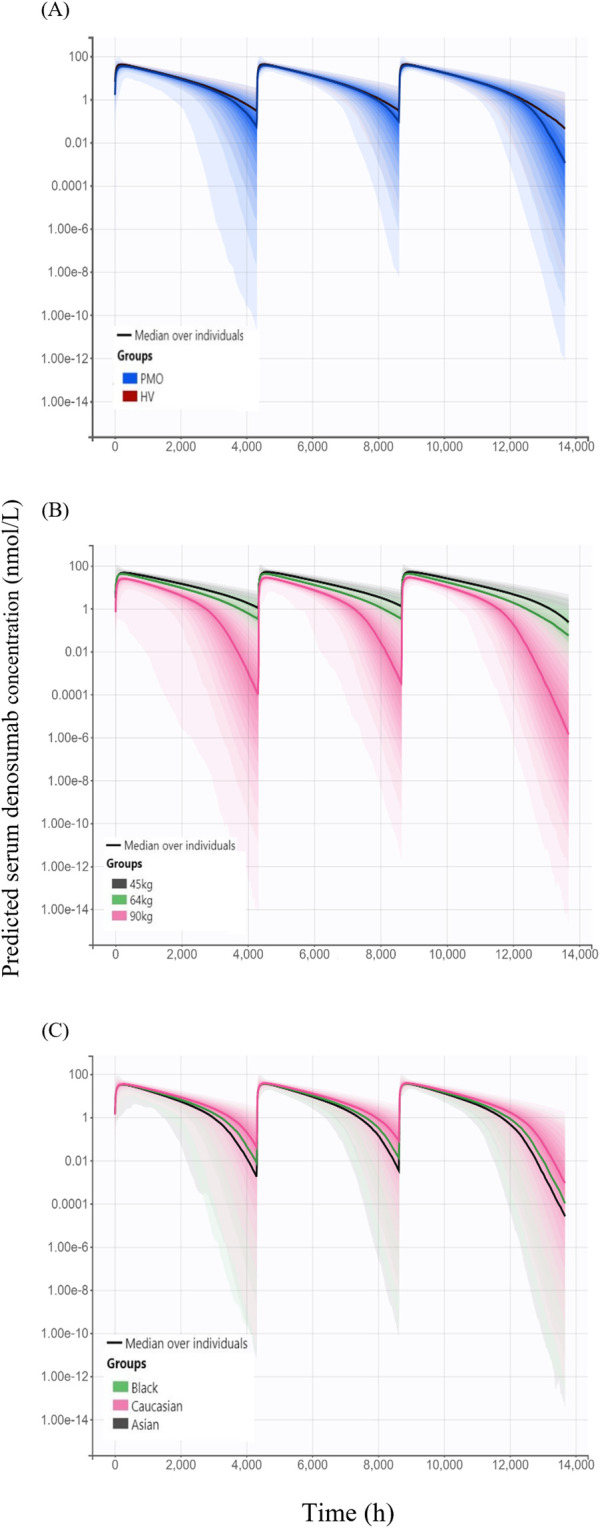
Simulated serum concentration–time profiles following three subcutaneous administrations of 60 mg denosumab at 6-month intervals, stratified by covariates **(A)** Comparison of simulated profiles between study cohorts: healthy volunteers (HV, Phase I) and patients with postmenopausal osteoporosis (PMO, Phase III); **(B)** Effect of body weight (45 kg, 64 kg, and 90 kg) on denosumab exposure; **(C)** Effect of race (Caucasian, Black, and Asian) on denosumab exposure; Solid lines represent the median concentrations across simulated individuals, and shaded areas represent the 95% prediction intervals, reflecting inter-individual variability in concentration-time profiles.

Simulated denosumab exposure (steady-state AUC) across significant covariate subgroups are shown in Figure 6. The simulated AUC was approximately 4% lower in Phase III PMO patients compared to that in Phase I healthy subjects (Figure 6A). When stratified by body weight–represented by 45 kg (low), 64 kg (median), and 90 kg (high) the AUC was approximately 45% higher in the 45 kg group and 39% lower in the 90 kg group, relative to the 64 kg reference (Figure 6B). Additionally, simulated AUC values were approximately 11% and 19% lower in Black and Asian subjects, respectively, compared to Caucasians.

**FIGURE 7:**
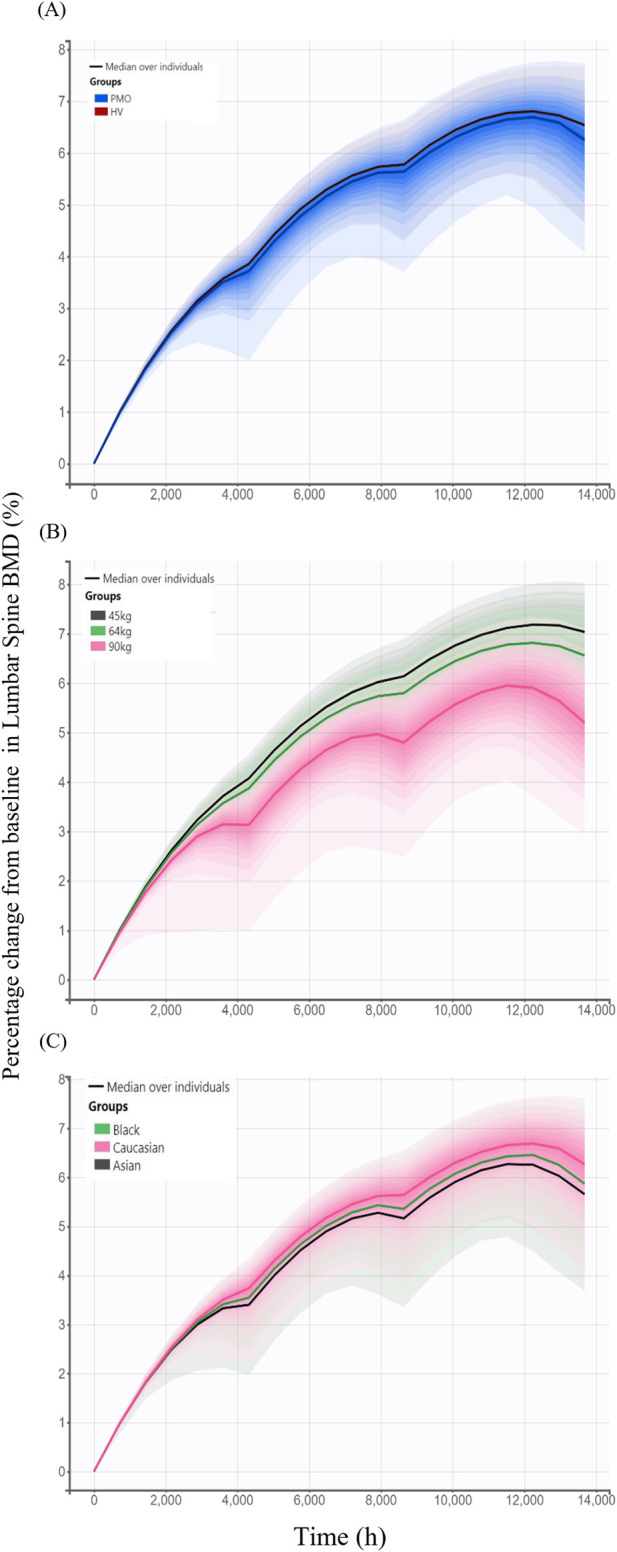
Simulated changes from baseline in lumbar spine bone mineral density over 18 months following three subcutaneous administrations of 60 mg denosumab at 6-month intervals stratified by covariates. **(A)** Comparison between study cohorts: healthy volunteers (HV, Phase I) and patients with postmenopausal osteoporosis (PMO, Phase III); **(B)** Effect of body weight (45 kg, 64 kg, and 90 kg); **(C)** Effect of race (Caucasian, Black, and Asian); Solid lines represent the median profiles across simulated individuals, and shaded areas represent the 95% prediction intervals, reflecting inter-individual variability.

Model-based simulations were performed to evaluate how difference in denosumab exposure across covariates influences PD responses, measured by changes from baseline in lumbar spine BMD. The percentage change from baseline in lumbar spine BMD following 6-month treatment intervals over 18 months was slightly higher in the Phase I healthy subjects (6.65%) than in the Phase III PMO patients (6.45%) (Figure 7A). Simulated changes from baseline in lumbar spine BMD by weight groups were 7.11%, 6.66%, and 5.54% for the 45 kg, 64 kg and 90 kg groups, respectively (Figure 7B). Similarly, simulated changes from baseline in lumbar spine BMD were 6.45%, 6.15%, and 5.93% for Caucasian, Black, and Asian subjects, respectively (Figure 7C).

### 3.3 PK/PD comparison of SB16 and DEN

**FIGURE 8:**
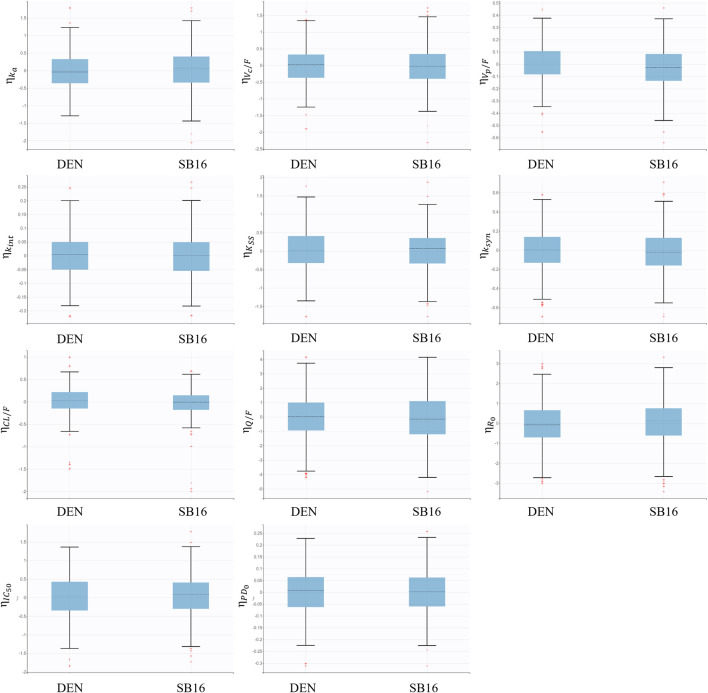
Boxplots for inter-individual variability of main fixed effect parameters stratified by treatment groups.

Treatment (SB16 vs. DEN) was not identified as a statistically significant covariate since decrease in OFV was observed during covariate screening. Consequently, it was not included in the final PK-PD model. Boxplots of the EBEs for key PK and PD parameters showed that the distributions of ETAs were highly comparable between the two treatment groups, with no significant difference observed (Figure 8).

**FIGURE 9:**
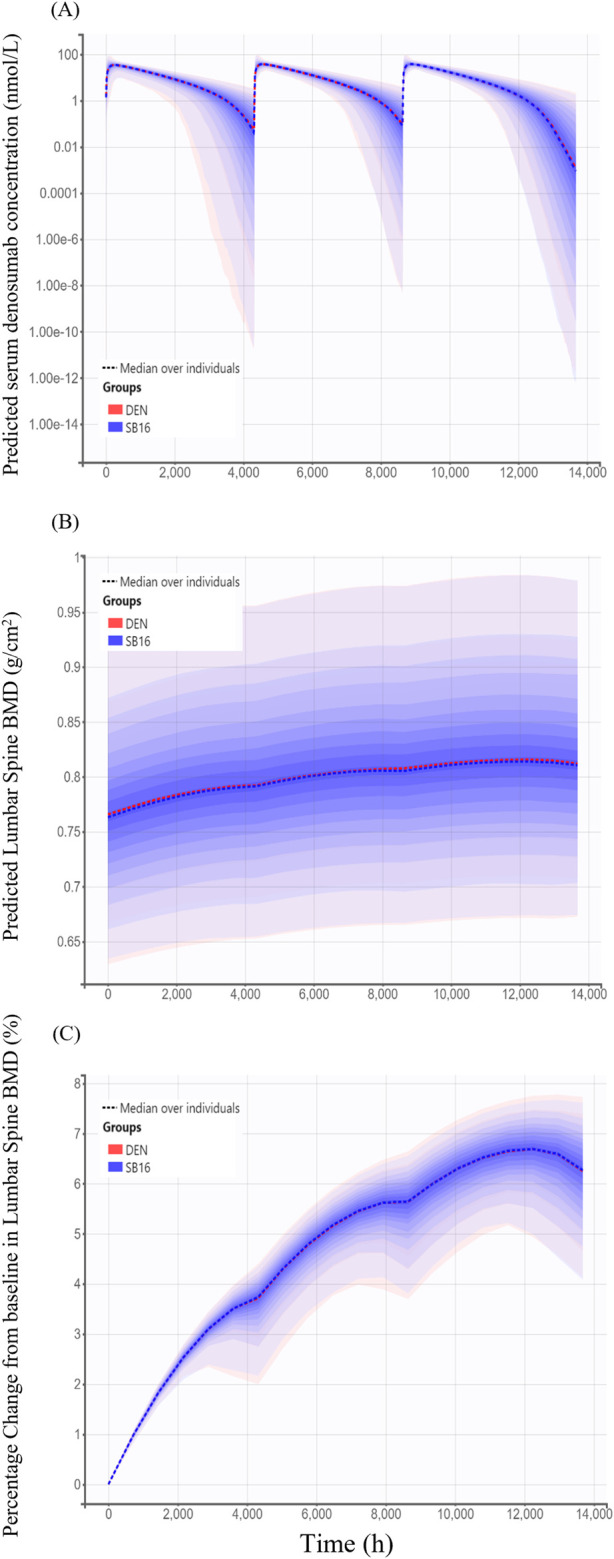
Simulated time profiles comparing SB16 and DEN after three subcutaneous administrations of 60 mg denosumab at 6-month intervals **(A)** Predicted serum concentration–time profiles; **(B)** Predicted lumbar spine bone mineral density (BMD); **(C)** Predicted percentage change from baseline in lumbar spine BMD. Solid lines represent the median profiles across simulated individuals, and shaded areas represent the 95% prediction intervals, reflecting inter-individual variability.

For treatment-specific simulation, the treatment group was intentionally included as a covariate in the final PK-PD model. The implemented CL/F ratio of SB16 to DEN was 0.9982, indicating a negligible difference between treatments. As shown in Figure 9, the time–concentration profile, time-lumbar spine BMD profile, and percentage change from baseline in lumbar spine BMD over time were nearly identical between the biosimilar and the reference product. A comparison of secondary PK and PD parameters demonstrated highly similar results between the two treatment groups (Table 5). To further confirm the robustness of this comparability, an additional simulation was conducted using individual PK and PD parameters estimated specifically in postmenopausal women with osteoporosis. The resulting profiles remained consistent with the primary findings and are provided in Supplementary Figure S1.

**TABLE 5:** Comparison of simulated PK and PD results between SB16 and DEN.

Parameters	SB16	DEN
	Median (90% prediction interval)	
C_max_ (nmol/L)	40.66 (26.90–77.50)	40.29 (26.04–80.45)
T_max_ (h)	259 (52–530)	267 (59–505)
AUC_τ,ss_ (nmol/L/h)	55,040 (33,625–85,011)	53,731 (33,706–87,790)
Change from baseline in lumbar spine BMD (%)	6.59 (4.90–7.52)	6.59 (4.96–7.58)

C_max_: maximum concentration; T_max_: time to maximum concentration; AUC_τ, ss_: area under the curve over a dosing interval at steady-state; BMD: bone mineral density; DEN: reference denosumab.

## 4 Discussion

Population PK/PD analyses were conducted using combined data from a comparative clinical PK study and a comparative efficacy and safety study to characterize the PK/PD of denosumab and compare SB16, a biosimilar with EU/US-DEN, the reference product. Consistent with a previous report for reference denosumab (Sutjandra et al., 2011), a two-compartment TMDD model based on QSS approximation and first-order absorption adequately described the PK data for SB16 and EU/US-DEN. In addition, an indirect response PD model with a maximal inhibitory function captured the treatment effect on lumbar spine BMD following administration.

RANKL concentrations data were not available for the current analysis; therefore, the developed QSS-TMDD model leveraged prior knowledge of the mechanistic interaction between denosumab and RANKL, alongside both linear and target-mediated drug clearance pathways. This approach allowed for the estimation of physiologically plausible, target-specific model parameters that were overall consistent with the reported values for therapeutic monoclonal antibodies (Gibiansky and Gibiansky, 2019). However, drug concentration data alone were insufficient to capture the turnover rate of the target (k_deg_) and drug-target complex (k_int_). The estimated K_SS_ (K_D_ + k_int_/k_on_) value of 1.56 nM was higher than the reported *in vitro* K_D_ of 3 pM (Arthur et al., 2012), with low variability (<25%RSE). The absence of target concentration data limits the precise estimation of the parameters related to pharmacological target dynamics in a full TMDD framework.

High shrinkage was observed in TMDD-related parameters (R_0_, k_int_, and K_SS_), likely due to the limited informativeness of the dataset for estimating receptor-mediated dynamics. In particular, the absence of receptor concentration data is a known factor that impairs the identifiability of TMDD parameters and leads to increased shrinkage (Gibiansky et al., 2008). Shrinkage was also high for the IC_50_ parameter in the PD model, which can be attributed to the sparse sampling of BMD, limiting the ability to characterize the inflection point of the exposure–response relationship. While these observations reflect limitations in parameter identifiability, they do not indicate model misspecification and are not expected to compromise the structural adequacy or overall interpretability of the final model (Savic and Karlsson, 2009; Xu et al., 2012). To address potential diagnostic bias due to high shrinkage, individual predictions were generated using Monolix’s conditional distribution approach, which provides a more reliable basis for GOF and covariate evaluations than EBE (Monolix Suite Documentation, 2024).

As changes in BMD reflect the net outcome of physiological bone remodeling, the delayed response to denosumab treatment was mechanistically characterized using an indirect response model, which is commonly applied to describe the endogenous turnover system (Felmlee et al., 2012). To reflect the known mechanism of action by which denosumab inhibits osteoclast activation, the drug was modeled to suppress the natural bone resorption rate constant (k_out_), thereby increasing BMD (Yee and Raje, 2012). A Hill coefficient was incorporated to allow flexible PD modeling (Gabrielsson and Hjorth, 2016). The IC_50_ value was estimated to be 6.92 nmol/L, representing the concentration at which denosumab achieves 50% of its maximal inhibitory effect on BMD degradation.

Covariate analysis revealed that the study population (HV vs. PMO patients) influences drug absorption and intercompartmental clearance (Q/F). The k_a_ was estimated to be 0.014 h^-1^ in HV and 0.0078 h^-1^ in PMO patients, corresponding to an absorption half-life of 2.1 and 3.7 days, respectively. This difference may be partially attributed to overall health status and age-related reductions in lymph flow rate, with younger individuals exhibiting higher lymphatic flow and, consequently, faster absorption rate (Thomas and Balthasar, 2019). Given that denosumab is indicated for the treatment of osteoporosis, the estimated absorption half-life of 3.7 days along with an IIV of 56.57% in this model is consistent with the previously reported human value of 3.3 days (Sutjandra et al., 2011). The Q/F was estimated at 1.13 L/h and 0.20 L/h in HV and PMO, respectively. However, the effects of the study population on k_a_ and Q/F are translated into <5% difference in the simulated AUC and BMD outcomes. As expected, baseline target concentrations were higher in PMO patients (15.23 nM) than in healthy individuals (0.98 nM) (Lewiecki, 2011), but this difference had minimal influence on the estimation of target dynamics, owing to the low variability (<25%RSE) associated with R_0_ and k_int_.

The estimated central and peripheral volumes of distribution (V_C_/F and V_P_/F) were 1.58 L and 6.06 L, respectively, and were modeled as functions of body weight, estimated power exponents of 1.50 for V_C_/F and 0.52 for V_P_/F. Although the estimated V_C_ was lower than that of plasma volume, the steady-state volume of distribution encompassing both central and peripheral compartments was 7.64 L. This value falls within the range reported in the Phase I study (SB16-1001) and aligns with the known V_SS_ range of 2.9–20 L for therapeutic monoclonal antibodies (Kuester et al., 2006; Roskos et al., 2004; Dirks and Meibohm, 2010).

The relatively small value of V_C_/F may be attributed to structural limitations in the absorption model. As previously reported, misspecification of the absorption process can lead to biased estimation of distribution parameters such as V_C_/F and bioavailability (Therapeutic Goods Administration, 2011; Wade et al., 1993). Given the complexity of subcutaneous absorption and relatively sparse data points during the absorption phase in this study, the estimated V_C_/F likely reflects limitations in the absorption model rather than a physiologically meaningful volume (Dirks and Meibohm, 2010).

Body weight and race were identified significant covariates on CL/F. Model-based simulations adjusting for these covariates showed that the AUC was approximately 45% higher in individuals weighing 45 kg and 39% lower in those weighing 90 kg relative to the 64 kg reference group. The predicted AUCs were 11% and 19% lower in Black and Asian subjects, respectively, relative to Caucasians. Despite these differences, no clinically meaningful difference was observed in PD outcomes; lumbar spine BMD responses remained within a narrow range across subgroups 7.11%, 6.66%, and 5.54% by weight; 6.45%, 6.15%, and 5.93% by race. Furthermore, no patient characteristics were identified as significant covariates influencing the PD parameters associated with changes in lumbar spine BMD. These findings are consistent with clinical data demonstrating comparable improvement in BMD regardless of race or BMI, supporting the current recommended dosage (European Medicines Agency, 2010; Jaber et al., 2020; European Medicines Agency, 2024c).

For similarity assessment of biosimilar SB16 and the reference product, EU- and US-sourced reference products were analyzed as a single pooled-reference group rather than as separate comparators. This approach is justified, as the same pivotal clinical data were used to support the approval of the originator product in both regions (European Medicines Agency, 2010; U.S. Food and Drug Administration, 2013). Furthermore, SB16 has already been approved in both the EU and US following rigorous similarity evaluations, including bridging studies (U.S. Food and Drug Administration, 2025; European Medicines Agency, 2024a). Two approaches were employed to assess the similarity. First, the model-generated plots showed that CL/F and I_max_ exhibited similar ETA distributions between SB16 and DEN. Although the treatment group was not identified as a significant covariate, including this factor as a predictor for CL/F in the model yields a CL/F the ratio of was 0.9982 between groups, with a standard error close to zero. Second, model-based simulations including treatment group as a covariate on CL/F showed that the change from baseline in lumbar spine BMD at 18 months for both SB16 and DEN was 6.59, with overlapping prediction intervals. The results align with BMD changes observed in clinical trials −6.5% increase at 24 months (Bone et al., 2008) and a mean increase of 3.0%–6.7% at 12 months in osteoporotic women (McClung et al., 2006). The modeling-based approach provides mechanistic and quantitative evidence of biosimilarity based on understanding inter-individual variability in PK/PD.

Although lumbar spine BMD has been accepted as a primary endpoint for assessing similarity in therapeutic efficacy in osteoporosis treatment (Jeka et al., 2024), our analysis results showed a wide range of AUC values (61%–145%) across subgroups corresponded to a narrower BMD response range (84%–107%), suggesting a plateau effect at the tested dose. This observation likely reflects the inherently slow biological kinetics of bone remodeling; BMD changes emerge gradually over months following antiresorptive therapy due to the low turnover rate of bone tissue. Consequently, BMD may not adequately capture modest or short-term differences in drug exposure, particularly in comparisons between the products expected to be highly similar.

To address this limitation, more responsive PD biomarkers may be required to improve sensitivity in biosimilarity assessments. For example, serum C-terminal telopeptide (CTX), a key marker of bone resorption, has been shown to decrease significantly within several days of denosumab administration, whereas BMD changes take several months to become apparent (McClung et al., 2006). In line with this, our ongoing research focuses on developing an integrated PK/PD model that incorporates denosumab PK, CTX dynamics, and subsequent BMD changes from baseline to characterize the relationships among these factors and to enable early prediction and monitoring of treatment effects.

In conclusion, this population PK/PD analysis of SB16 and reference denosumab, using data from studies involving healthy subjects and PMO patients, well characterized the PK/PD profile of denosumab. This is achieved by a two-compartment QSS-TMDD model integrated with an inhibitory effect function in the indirect response PD model. No key patient variables were found to significantly influence the PD response of the drug, and any observed difference in drug exposure among patient subgroups was considered to have negligible clinical relevance. Furthermore, model-based simulations demonstrated PK/PD similarity between SB16 and reference denosumab, supporting the potential for SB16 to be substituted for the reference product in treatment of osteoporosis.

## Data Availability

The original contributions presented in the study are included in the article/Supplementary Material. Any further inquiries can be directed to the corresponding author.

## Abbreviations

DEN, denosumab; PK, pharmacokinetics; PMO, postmenopausal women with osteoporosis; PD, pharmacodynamics; HV, healthy volunteers; BMD, bone mineral density; CL, clearance; TMDD, target-mediated drug disposition; QSS, quasi-steady state; RANKL, receptor activator of nuclear factor kappa-Β ligand; BTM, bone turnover markers; SC, single subcutaneous; ECLIA, electrochemiluminescence immunoassay; LLOQ, lower limit of quantification; BLQ, below the limit of quantification; IP, investigational product; EOS, end of study; ET, early termination; DXA, dual-energy X-ray absorptiometry; IIV, Interindividual variability; LRT, likelihood ratio test; GOF, goodness-of-fit; RV, residual variability; OFV, objective function value; VPC, visual predictive checks; PI, prediction interval.
