# Supplementary material for: Population pharmacokinetics/pharmacodynamics analysis confirming biosimilarity of SB16 to reference denosumab
Source: Front Pharmacol. 2025 Aug 19;16:1631034. doi: 10.3389/fphar.2025.1631034 (PMC12403037; doi:10.3389/fphar.2025.1631034)
Supplement: Supplementary file 1 [file Supplementaryfile1.docx]

Supplementary Material

Table S1 Demographic Characteristics by Treatment Group (Randomised Set, SB16-3001 study)

| **Characteristics** | | **SB16 N = 225** | | **Prolia** | | | | | | **Total N = 457** |
| --- | --- | --- | --- | --- | --- | --- | --- | --- | --- | --- |
|  |  |  |  | **Overall N = 232** | | **SB16 N = 100^a^** | | **Prolia N = 101^a^** | |  |
| Age (years) |  | |  | |  | |  | |  | |
| Mean | 66.5 | | 66.3 | | 65.8 | | 66.4 | | 66.4 | |
| SD | 5.87 | | 6.03 | | 5.73 | | 6.05 | | 5.95 | |
| Age group, n (%) |  | |  | |  | |  | |  | |
| < 65 years | 89 (39.6) | | 95 (40.9) | | 39 (39.0) | | 44 (43.6) | | 184 (40.3) | |
| ≥ 65 years | 136 (60.4) | | 137 (59.1) | | 61 (61.0) | | 57 (56.4) | | 273 (59.7) | |
| Race, n (%) |  | |  | |  | |  | |  | |
| Asian | 18 (8.0) | | 23 (9.9) | | 10 (10.0) | | 11 (10.9) | | 41 (9.0) | |
| White | 207 (92.0) | | 208 (89.7) | | 89 (89.0) | | 90 (89.1) | | 415 (90.8) | |
| Other | 0 (0.0) | | 1 (0.4) | | 1 (1.0) | | 0 (0.0) | | 1 (0.2) | |
| Ethnicity, n (%) |  | |  | |  | |  | |  | |
| Hispanic or Latino | 0 (0.0) | | 1 (0.4) | | 0 (0.0) | | 0 (0.0) | | 1 (0.2) | |
| Other | 225 (100.0) | | 231 (99.6) | | 100 (100.0) | | 101 (100.0) | | 456 (99.8) | |
| Country, n (%) |  | |  | |  | |  | |  | |
| Czech Republic | 59 (26.2) | | 59 (25.4) | | 26 (26.0) | | 28 (27.7) | | 118 (25.8) | |
| Denmark | 5 (2.2) | | 6 (2.6) | | 2 (2.0) | | 2 (2.0) | | 11 (2.4) | |
| Republic of Korea | 18 (8.0) | | 23 (9.9) | | 10 (10.0) | | 11 (10.9) | | 41 (9.0) | |
| Lithuania | 13 (5.8) | | 12 (5.2) | | 4 (4.0) | | 5 (5.0) | | 25 (5.5) | |
| Poland | 130 (57.8) | | 132 (56.9) | | 58 (58.0) | | 55 (54.5) | | 262 (57.3) | |
| Weight (kg) |  | |  | |  | |  | |  | |
| Mean | 64.01 | | 62.50 | | 61.33 | | 62.86 | | 63.24 | |
| SD | 9.940 | | 9.443 | | 9.299 | | 9.514 | | 9.709 | |
| Height (cm) |  | |  | |  | |  | |  | |
| Mean | 159.58 | | 158.54 | | 158.06 | | 158.70 | | 159.05 | |
| SD | 6.426 | | 6.220 | | 6.048 | | 6.191 | | 6.337 | |
| BMI (kg/m^2^) | | | | | | | | | | |
| Mean | 25.17 | | 24.86 | | 24.55 | | 24.95 | | 25.01 | |
| SD | 3.829 | | 3.462 | | 3.438 | | 3.407 | | 3.646 | |
| BMI level, n (%) | | | | | | | | | | |
| < 25 kg/m^2^ | 117 (52.0) | | 132 (56.9) | | 60 (60.0) | | 58 (57.4) | | 249 (54.5) | |
| ≥ 25 kg/m^2^ | 108 (48.0) | | 100 (43.1) | | 40 (40.0) | | 43 (42.6) | | 208 (45.5) | |

N = total number of subjects in the Randomised Set in each treatment group; BMI = body mass index; n = number of subjects with available data within each category; SD = standard deviation

Age was calculated as the difference in years of informed consent form and birth year obtained.

BMI (kg/m2) was calculated using baseline weight and height at Screening.

Percentages were based on the number of subjects in the Randomised Set.

a Based on subjects who had re-randomisation at Month 12, Prolia+SB16 and Prolia+Prolia may not add up to Prolia Overall.


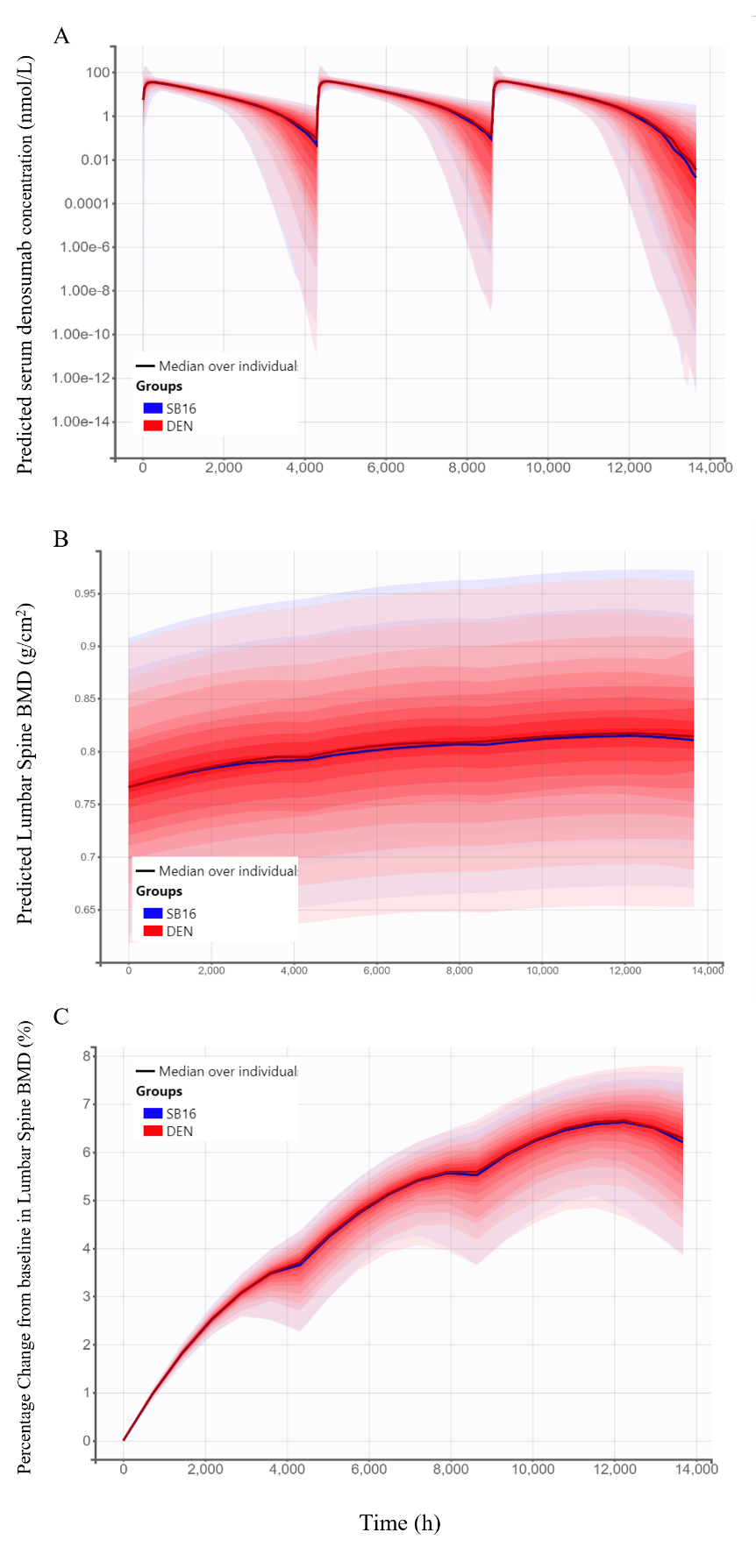


Figure S1 Simulated time profiles comparing SB16 and DEN after three subcutaneous administrations of 60 mg denosumab at 6-month intervals, using individual PK/PD parameters sampled from the conditional distribution estimated by the final model.

(A) Predicted serum concentration–time profiles; (B) Predicted lumbar spine bone mineral density (BMD); (C) Predicted percentage change from baseline in lumbar spine BMD. Solid lines represent the median profiles across simulated individuals, and shaded areas indicate the 95% prediction intervals, reflecting inter-individual variability.
